# Dietary apigenin potentiates the inhibitory effect of interferon-α on cancer cell viability through inhibition of 26S proteasome-mediated interferon receptor degradation

**DOI:** 10.3402/fnr.v60.31288

**Published:** 2016-06-28

**Authors:** Sheng Li, Li-juan Yang, Ping Wang, Yu-jiao He, Jun-mei Huang, Han-wei Liu, Xiao-fei Shen, Fei Wang

**Affiliations:** 1 Chengdu Institute of Biology, Chinese Academy of Sciences, Chengdu, China; 2 School of Chinese Pharmacy, Chengdu University of Traditional Chinese Medicine, Chengdu, China; 3 Ningbo Entry-Exit Inspection and Quarantine Bureau Technical Center, Ningbo, China; 4 Sichuan Translational Medicine Research Hospital, Chinese Academy of Sciences, Chengdu, China

**Keywords:** apigenin, interferon, proteasome, STAT, ubiquitination

## Abstract

**Background:**

Type I interferons (IFN-α/β) have broad and potent immunoregulatory and antiproliferative activities. However, it is still known whether the dietary flavonoids exhibit their antiviral and anticancer properties by modulating the function of type I IFNs.

**Objective:**

This study aimed at determining the role of apigenin, a dietary plant flavonoid abundant in common fruits and vegetables, on the type I IFN-mediated inhibition of cancer cell viability.

**Design:**

Inhibitory effect of apigenin on human 26S proteasome, a known negative regulator of type I IFN signaling, was evaluated *in vitro*. Molecular docking was conducted to know the interaction between apigenin and subunits of 26S proteasome. Effects of apigenin on JAK/STAT pathway, 26S proteasome-mediated interferon receptor stability, and cancer cells viability were also investigated.

**Results:**

Apigenin was identified to be a potent inhibitor of human 26S proteasome in a cell-based assay. Apigenin inhibited the chymotrypsin-like, caspase-like, and trypsin-like activities of the human 26S proteasome and increased the ubiquitination of endogenous proteins in cells. Results from computational modeling of the potential interactions of apigenin with the chymotrypsin site (β5 subunit), caspase site (β1 subunit), and trypsin site (β2 subunit) of the proteasome were consistent with the observed proteasome inhibitory activity. Apigenin enhanced the phosphorylation of signal transducer and activator of transcription proteins (STAT1 and STAT2) and promoted the endogenous IFN-α-regulated gene expression. Apigenin inhibited the IFN-α-stimulated ubiquitination and degradation of type I interferon receptor 1 (IFNAR1). Apigenin also sensitized the inhibitory effect of IFN-α on viability of cervical carcinoma HeLa cells.

**Conclusion:**

These results suggest that apigenin potentiates the inhibitory effect of IFN-α on cancer cell viability by activating JAK/STAT signaling pathway through inhibition of 26S proteasome-mediated IFNAR1 degradation. This may provide a novel mechanism for increasing the efficacy of IFN-α/β.

Considerable attention has been devoted to identifying plant-derived dietary agents that could be developed as chemopreventives. One such agent is apigenin, a naturally occurring plant flavonoid (4’, 5, 7-trihydroxyflavone) abundant in common fruits and vegetables, including parsley, onions, oranges, tea, chamomile, wheat sprouts, and some seasonings. Apigenin exerts anti-inflammatory, antioxidant, and anticarcinogenic effects by targeting multiple cellular factors (1). Previously, apigenin has been reported to potently inhibit the chymotrypsin-like activity of 26S proteasome and induce apoptosis in human leukemia cells, breast cancer cells, and other cancer cell lines (2–4). The inhibitory effect of apigenin and other natural flavonoids, such as (-)-epigallocatechin-3-gallate (EGCG), quercetin, chrysin, and resveratrol, on 26S proteasome may partly explain their beneficial effects in cancer prevention (5). However, the differential effects of apigenin on the three peptidase-like activities of the human 26S proteasome are unknown. These include caspase-like activity (β1 subunit), trypsin-like activity (β2 subunit)**, and chymotrypsin-like activity (β5 subunit), each of which has a distinct role in protein breakdown. These differential effects may influence the development of antineoplastic drugs (6, 7).

Type I interferons (IFN-α/β) are pleiotropic cytokines with broad and potent immunoregulatory and antiproliferative activities. IFNs mediate their effects by binding to cell-surface IFN receptors (IFNAR1/2), activating Janus kinases (Jak1, Tyk2), and subsequently phosphorylating signal transducer and activator of transcription proteins (STAT1, STAT2), which translocate into the nucleus and recognize a specific interferon α-stimulated response element (ISRE) motif in the promoter region of certain transcription genes (8). Type I IFNs have been clinically used for the treatment of various viral or cancerous diseases, such as hepatitis B/C, multiple sclerosis, leukemia, and melanoma. However, the side effects, such as neuropsychiatric and hematologic toxicities, which appear to be directly related to the dose and duration of IFN therapy, and drug tolerance severely limit their clinical use (9). Thus, chemical compounds that can mimic or enhance the effect of type I IFNs on the JAK/STAT signaling pathway have potential to be developed as next-generation antiviral and anticancer drugs. Some compounds have been identified through cell-based ISRE reporter screening to activate JAK/STAT signaling and exhibit antiviral or antiproliferative effects through different mechanisms, including suppression of the cyclic AMP-protein kinase A (PKA) – Src homology phosphatase 2 domain containing phosphatase (SHP2) pathway, inhibition of pyrimidine biosynthesis, or activation of type I IFN receptors (10–12). Thus, it is of interest to further investigate whether dietary compounds can exert beneficial effect on JAK/STAT signaling, the major system in humans for the prevention of infection and carcinogenesis.

The ubiquitin/26S proteasome pathway plays an important role in the negative regulation of IFN signaling. The binding of IFN to IFNAR1 induces the phosphorylation and ubiquitination of IFNAR1, which leads to the subsequent lysosomal degradation of ligand–IFNAR1 complex (13, 14). Phosphorylated Jak2 is polyubiquitinated and degraded by the 26S proteasome, which is regulated by the suppressor of cytokine signaling 1 (SOCS-1) (15). Phosphorylated STAT1 is also recognized by the F-box E3 ligase SCF^βTRCP^ for ubquitination and 26S proteasomal degradation (16). Thus, inhibition of 26S proteasome may be a new therapeutic means to enhance the efficacy of IFNs and reduce their side effects.

Apigenin was identified as an activator of the JAK/STAT pathway and was found to increase the endogenous antiviral gene expression regulated by the type I IFNs (17). However, the mechanism by which apigenin acts on the JAK/STAT pathway is still unknown. In this study, we investigated the inhibition of proteasome and potentiation of type I IFN-induced JAK/STAT signaling by apigenin in detail.

## Materials and methods

### Reagents and plasmids

Apigenin (purity ≥99%, HPLC) was purchased from Must Biotechnology Co., Ltd. (Chengdu, China). IFN-α (recombinant human IFN-α2a) and IFN-β (recombinant human IFN-β1b) were purchased from ProSpec-Tany Techno Gene, Ltd. (Shanghai, China). Bortezomib, anti-ubiquitin antibody, and protein A/G agarose were purchased from Santa Cruz Biotechnology (Santa Cruz, CA). MG132 was purchased from Sigma-Aldrich (Shanghai, China). The purified human 26S proteasome, Ac-Arg-Leu-Arg-AMC, and Suc-Leu-Leu-Val-Tyr-AMC were purchased from Enzo Life Sciences (Farmingdale, NY). Z-Nle-Pro-Nle-Asp-aminoluciferin was purchased from Promega (Beijing, China). Anti-phospho-STAT1, anti-STAT1, anti-phospho-STAT2, anti-STAT2, and anti-GAPDH antibodies were purchased from Abcam (Cambridge, MA). Anti-IFNAR1 and anti-Ki67 antibodies were purchased from Proteintech (Chicago, IL). The 37-amino-acid long C-terminus of mouse ornithine decarboxylase (cODC) was amplified through a plasmid containing full-length mouse ODC (kindly provided by Prof. Philip Coffino, University of California San Francisco). The pCIneo-luciferase-cODC plasmid was obtained by subcloning the cODC fragment into a pCIneo-luciferase plasmid. This plasmid was constructed by cloning the luciferase gene from a pGL4.26 vector (Promega) into the pCI-neo mammalian expression vector (Promega). pCMV-ubiquitin plasmid was constructed by cloning the full-length coding sequence of human ubiquitin cDNA into pCMV 7.1 expression vector (Sigma). TUNEL apoptosis assay kit, Alexa Fluor 555-labeled anti-rabbit IgG and 4’, 6-diamidino-2-phenylindole (DAPI) were obtained from Beyotime (Haimen, China).

### Cell culture

Human embryonic kidney 293A (HEK293A) cells (Qbiogene, Carlsbad, CA) and human cervical cancer HeLa cells (American Type Culture Collection, Manassas, VA) were maintained in Dulbecco's Modified Eagle Medium (DMEM; Invitrogen, Carlsbad, CA) containing 10% fetal calf serum (Invitrogen), and 1% penicillin/streptomycin at 37°C in a 5% CO_2_ atmosphere. The HEK293A-luciferase-cODC cell line and HepG2-ISRE-Luc2 cell line were established and maintained as previously reported (17, 18).

### Measurement of peptidase activity

The purified human 26S proteasome (0.1 µg) was incubated with or without different concentrations of apigenin in 100 µL assay buffer (50 mM Tris-HCl, pH 7.5) and 40 µM fluorogenic peptide substrate Suc-Leu-Leu-Val-Tyr-AMC (for the proteasomal chymotrypsin-like activity), Ac-Arg-Leu-Arg-AMC (for the proteasomal trypsin-like activity), or Z-Nle-Pro-Nle-Asp-aminoluciferin (for the proteasomal caspase-like activity) for 2 h at 37°C. After incubation, the fluorescence was measured with a Thermo Scientific Varioskan Flash multimode reader.

### Computational binding simulation

Molecular docking was carried out as previously reported with minor modifications (2). The crystal structure of eukaryotic yeast 20S proteasome was obtained from the Protein Database (Ref. number 1JD2) and used for all the docking studies presented here. The molecular docking simulation was performed and analyzed using AutoDock 4.2 and AutoDock Vina (19). AutoDock 4.2, a Lamarckian genetic algorithm method implemented in the program suite, was employed to identify appropriate binding modes and conformation of the ligand molecules. Docking pose was chosen based on two criteria. First, the proximity to the N-terminal threonine should lie in 3–4Å (a distance suitable for nucleophilic attack). Second, placement of the A–C ring system of the molecule should be within the S1 hydrophobic pocket. Of the orientations/conformations that fit these two criteria, the docked structure of lowest docked free energy was chosen. The output from AutoDock and all modeling studies as well as images was rendered with PyMOL, which was used to calculate the distances of hydrogen bonds as measured between the hydrogen and its assumed binding partner (20).

### Western blotting

Cells were lysed in RIPA buffer and the lysates were subjected to sodium dodecyl sulfate-polyacrylamide gel electrophoresis. The proteins were blotted onto nitrocellulose membranes, probed with each specific antibody and an appropriate peroxidase-conjugated secondary antibody, and detected by enhanced chemiluminescence (Amersham Biosciences, Piscataway, NJ). Protein concentration was measured with a BCA protein assay kit (Bestbio, Shanghai, China).

### Real-time quantitative reverse transcription-polymerase chain reaction

Total cellular RNA was isolated by TRIzol reagent, which was then reverse-transcribed using SuperScript III Reverse Transcriptase with oligo dT_18_ primer (Invitrogen). Equal amounts of complementary DNA were subjected to real-time quantitative PCR with the fluorescent dye SYBR Green I. The primer pairs used in the assay for IFN-induced double-stranded RNA-activated protein kinase (PKR), 2’,5’-oligoadenylate synthetase 1 (2’,5’-OAS1), and GAPDH were the following: 5’-GTTTGCTTCTCTGGCGGTCTT-3’/5’-GCCATTTCTTCTTCCCGTATCC-3’ (PKR), 5’-AGGTGGTAAAGGGTGGCTCC-3’/5’-ACAACCAGGTCAGCGTCAGAT-3’ (2’,5’-OAS1), 5’-TGCACCACCAACTGCTTAGC-3’/5’-GGCATGGACTGTGGTCATGAG-3’ (GAPDH). The quantity of PKR and 2’,5’-OAS1 mRNA was normalized to the endogenous reference (GAPDH) mRNA in the same samples.

### Immunoprecipitation

Cell lysates were prepared in ice-cold immunoprecipitation buffer (20 mM Tris-HCl, 200 mM NaCl, 1 mM EDTA, 0.5% Nonidet P-40, pH 8.0) with PMSF and protease cocktail inhibitors (Sigma). The mixture was centrifuged at 13,000×g for 15 min, cleared with normal immunoglobulin G coupled to agarose beads (protein A/G) for 1 h, incubated overnight at 4°C with IFNAR1 antibody, and then incubated with protein A/G agarose beads for 4 h. The precipitates were washed thrice in PBS buffer, centrifuged at 3,000×g for 3 min, resuspended in 5× loading buffer, and boiled for 5 min. The supernatants were loaded onto sodium dodecyl sulfate-polyacrylamide gel electrophoresis and immunoblotted with anti-ubiquitin antibody.

### Cell viability assay

HeLa cells were seeded in 96-well plates at a density of 5×10^3^ cells/well in 100 µL of media. Cultured cells were treated with apigenin or a combination of apigenin with IFN-α at the indicated concentrations. After 72 h, 10 µL of Alamar Blue reagent was added to the media and incubated for another 2–4 h until the development of pink color. The relative fluorescence intensity was measured using a Thermo Scientific Varioskan Flash multimode reader.

### Colony formation assay

The HeLa cells, at the logarithmic phase, were plated in 6-well plates at the density of 600 cells/well. Cells were treated with emodin in combination with IFN-α and allowed to grow for 12 days to form colonies. A colony was defined as a cluster of more than 50 cells. Then, cells were fixed with methanol and stained with 0.1% crystal violet solution for 20 min, and the colonies (>50 cells) were counted under microscope.

### Statistical analysis

Statistical analyses were performed with GraphPad Prism 5.0 software (GraphPad, La Jolla, CA). All experiments were repeated at least thrice. Representative results are presented. Data were compared by one-way ANOVA followed by Dunnett's post hoc test. The differences were considered statistically significant when *p*≤0.05.

## Results

### Identification of apigenin as an inhibitor of 26S proteasome

**Fig. 1:**
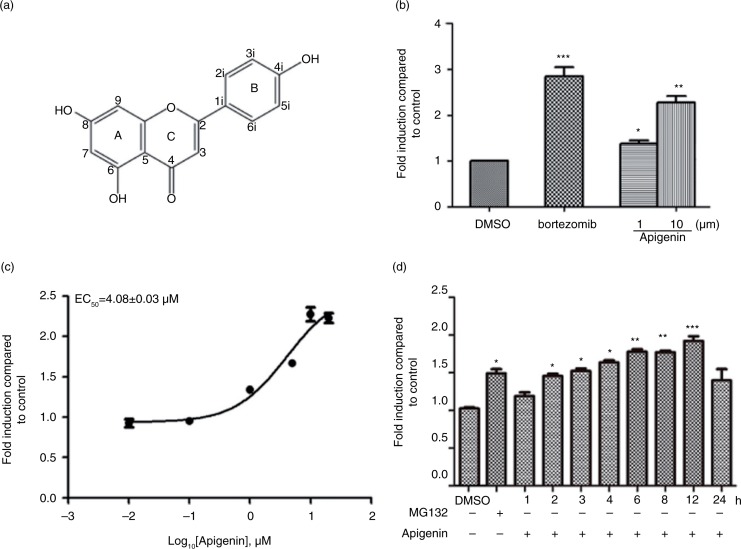
Identification of apigenin as an inhibitor of 26S proteasome in HEK293A-luciferase-cODC cells. (a) Chemical structure of apigenin. (b) HEK293A-luciferase-cODC cells were seeded in 96-well plates and treated in the presence of indicated concentrations of apigenin or bortezomib for 3 h. (c) Concentration–response curve for apigenin. The calculated EC_50_ value was 4.08 µM. (d) HEK293A-luciferase-cODC cells were seeded in 96-well plates and treated with 10 µM apigenin for the indicated time, or treated with 1 µM MG132 for 4 h. Results are representative of three separate experiments. The error bars represent the standard deviation of the measurements. (*) *p*<0.05, (**) *p*<0.01, (***) *p*<0.001 compared to the DMSO control.

To identify inhibitors of 26S proteasome, we established a HEK293A cell line stably transfected with pCIneo-luciferase-cODC plasmid (HEK293A-luciferase-cODC). The native ODC is recognized by 26S proteasome for degradation through a small conserved degradation tag (37 amino acids in mouse and human) at its C-terminus (cODC) without the need of ubiquitin modification (21). Using this ubiquitin-independent, 26S proteasome-specific cell-based assay, we screened our chemical library consisting of 1,431 natural products and synthetic analogues (17). After hit reconfirmation, apigenin was identified as potently increasing luciferase expression. The chemical structure of apigenin is illustrated in Fig. 1a. Bortezomib, a specific 26S proteasome inhibitor used in clinic for the treatment of multiple myeloma, also increased luciferase expression, confirming that HEK293A-luiferase-cODC cells specifically respond to inhibition of 26S proteasome (Fig. 1b). Apigenin at 1 and 10 µM increased luciferase activity 1.3- and 2.3-fold, respectively, over that of DMSO-treated control cells (Fig. 1b). The EC_50_ value for apigenin to inhibit luciferase-cODC degradation was 4.08 µM (Fig. 1c). Apigenin did not exhibit cytotoxicity at the concentrations examined (Supplementary Fig. 1). Promotion of luciferase-cODC expression by apigenin at 10 µM was first evident at 2 h and was sustained until 12 h, at which time luciferase-cODC expression increased by approximately 1.8-fold compared to that in the control (Fig. 1d). Similarly, a proteasomal inhibitor MG132 at 1 µM also promoted luciferase-cODC expression in a time-dependent manner (Supplementary Fig. 2). These results suggest that apigenin inhibits 26S proteasome to stabilize luciferase-cODC.

### Inhibitory effect of apigenin on 26S proteasome activity

**Fig. 2:**
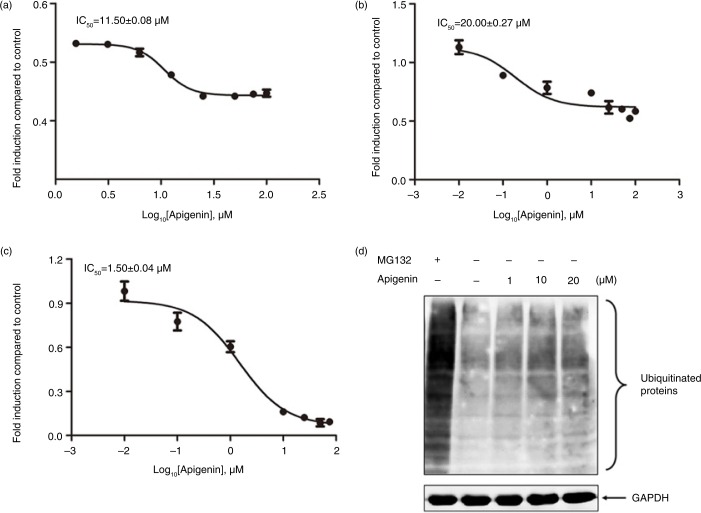
Inhibitory effect of apigenin on 26S proteasome activity. The purified human 26S proteasome (0.1 µg) was treated with different concentrations of apigenin and (a) 40 µM Suc-Leu-Leu-Val-Tyr-AMC (for the chymotrypsin-like activity), (b) 40 µM Ac-Arg-Leu-Arg-AMC (for the trypsin-like activity), (c) 40 µM Z-Nle-Pro-Nle-Asp-aminoluciferin (for the caspase-like activity) for 2 h at 37°C. (d) HeLa cells were treated with the indicated concentrations of apigenin or 10 µM MG132 for 3 h, and the cell lysates were probed with anti-ubiquitin antibody. GAPDH was used as an internal control.

Purified human 26S proteasome was used to examine whether apigenin directly inhibits 26S proteasome. As shown in Fig. 2a, apigenin inhibited the chymotrypsin-like activity of purified 26S proteasome, with an IC_50_ value of 11.5 µM. Apigenin inhibited the trypsin-like activity of 26S proteasome with an IC_50_ value of 20 µM (Fig. 2b). Apigenin also potently inhibited the caspase-like activity of 26S proteasome, with an IC_50_ value of 1.5 µM (Fig. 2c). To examine the effect of apigenin on endogenous protein ubiquitination, we treated cells with apigenin (1–20 µM) and the total cell lysates were probed with ubiquitin antibody. As shown in Fig. 2d, inhibition of 26S proteasome by MG132 treatment significantly increased the endogenous protein ubiquitination level. Apigenin also increased endogenous protein ubiquitination levels. These results indicate that apigenin inhibits the activity of 26S proteasome, which can lead to the accumulation of protein ubiquitination in cells.

### Molecular docking of apigenin on the proteasome subunits

**Fig. 3:**
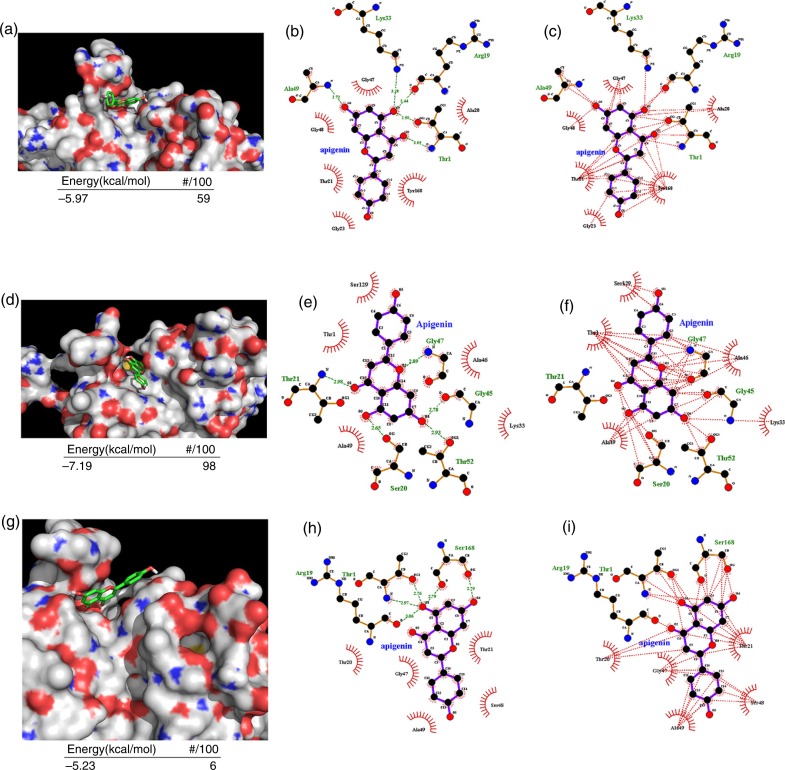
Molecular docking of apigenin on the proteasome subunits. (a–c) Apigenin was docked to β5 subunit of the proteasome. The energy of the inhibitory pose and the number of runs (out of 100) that adopted the inhibitory pose was shown below (a). The threonine catalytic residue and all amino acids in the S1 pocket of β5 subunit involved in the formation of H-bonds (b) and hydrophobic interactions (c) with apigenin are highlighted. (d–f) Apigenin was docked to β2 subunit of the proteasome. The energy of the inhibitory pose and the number of runs (out of 100) that adopted the inhibitory pose was shown below (d). The threonine catalytic residue and all amino acids in the S1 pocket of β2 subunit involved in the formation of H-bonds (e) and hydrophobic interactions (f) with apigenin are highlighted. (g–i) Apigenin was docked to β1 subunit of the proteasome. The energy of the inhibitory pose and the number of runs (out of 100) that adopted the inhibitory pose was shown below (g). The threonine catalytic residue and all amino acids in the S1 pocket of β1 subunit involved in the formation of H-bonds (h) and hydrophobic interactions (i) with apigenin are highlighted.

To investigate the nature of the interactions through which apigenin inhibits the activity of the proteasome, the compound was docked to the active site of the proteasome β1, β2, and β5 subunits, which are responsible for the caspase-like, trypsin-like, and chymotrypsin-like activity, respectively. AutoDock arranges its results by energy and clusters of solutions that adopt the same conformation (see Materials and Methods for details). As shown in Fig. 3a, apigenin adopted a conformation favorable for nucleophilic attack at the active site of the β5 subunit with an energy of −5.97 kcal/mol. To further investigate the favorable binding mode of apigenin to the proteasomal chymotrypsin active site, we analyzed hydrogen-bond (H-bond) formation and hydrophobic interactions between apigenin and the β5 subunit. There are three polar hydrogens and two carbonyl-oxygens on apigenin that are available for H-bonding (see Fig. 1a). It appears that two hydrogens and one carbonyl-oxygen are capable of actively participating in H-bonding with the side chains of Thr1, Arg19, Lys33, and Ala49 (Fig. 3b). The A–C ring system of apigenin was favorably oriented in the middle of the S1 hydrophobic pocket of the β5 subunit driven by hydrophobic interactions (Fig. 3c). Similarly, apigenin adopted a conformation favorable for nucleophilic attack at the active site of the β2 subunit with an energy of −7.19 kcal/mol (Fig. 3d). Two hydrogens in the A ring and two oxygens in the C ring of apigenin were capable of participating in H-bonding with the side chains of Thr21, Ser20, Thr52, Gly45, and Gly47 (Fig. 3e). The A–C ring of apigenin was favorably inserted within the S1 pocket of the β2 subunit by hydrophobic interactions (Fig. 3f). Apigenin also adopted a conformation favorable for nucleophilic attack at the active site of the β1 subunit with an energy of −5.32 kcal/mol (Fig. 3g). Two hydrogens in the A ring and two oxygens in the C ring of apigenin were capable of participating in H-bonding with the side chains of Thr1, Arg19, and Ser68 (Fig. 3h). The A–C ring of apigenin was also favorably inserted within the S1 pocket of the β1 subunit by hydrophobic interactions (Fig. 3i). These results suggest that apigenin can exhibit an orientation/conformation in the proximity to the N-terminal Thr1 of β1, β2, and β5 catalytic subunits making them subject to nucleophilic attack.

### Apigenin enhances the IFN-α/β-induced activation of the JAK/STAT pathway

**Fig. 4:**
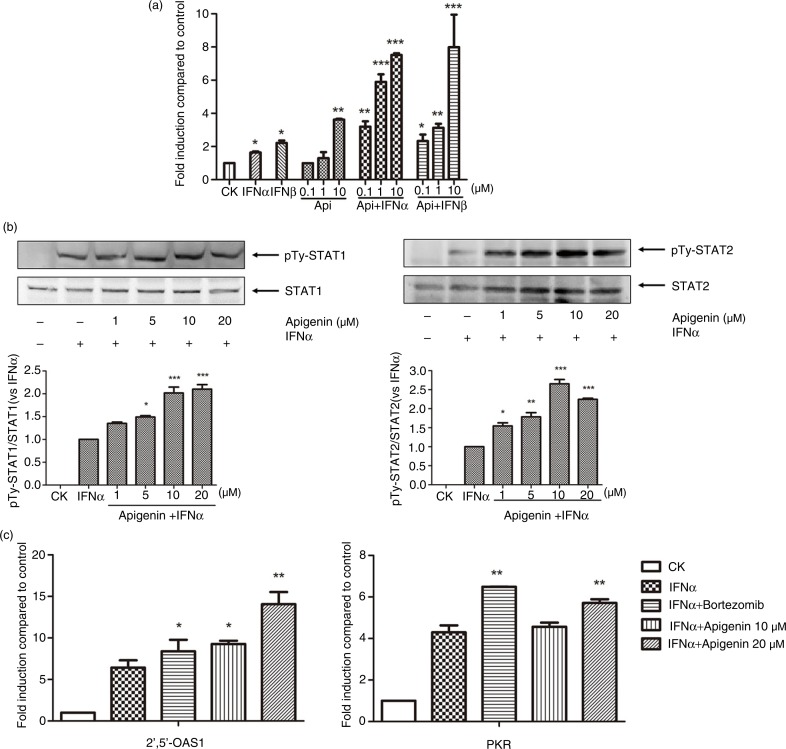
Apigenin enhances IFN-α/β-induced JAK/STAT activation. (a) HepG2-ISRE-Luc2 cells were seeded in 96-well plates (1×10^4^/well) and treated with the various concentrations of apigenin for 2 h, then with 200 U/mL IFN-α or IFN-β for 24 h. (b) HEK293A cells were incubated with indicated concentrations of apigenin for 2 h, then with 2000 U/mL IFN-α for another 1 h. The cell lysates were immunoblotted with phospho-STAT1 (Tyr701), STAT1, phospho-STAT2 (Tyr690), or STAT2 antibodies. The quantitative results are shown. (c) HEK293A cells were treated 200 U/mL IFN-α with or without 10 µM bortezomib or the indicated concentrations of apigenin for 24 h. Real-time quantitative reverse transcription-PCR was used to determine the mRNA expression of PKR or 2’,5’-OAS1. The result is presented as induction (n-fold) relative to basal levels in untreated cells. GAPDH was used as an internal control. (*) *p*<0.05, (**) *p*<0.01, (***) *p*<0.001 versus control (*n=*3).

Considering the important role of 26S proteasome in the attenuation of JAK/STAT pathway signaling, we hypothesize that apigenin may enhance the activation of JAK/STAT pathway by inhibiting 26S proteasome. To test this, we first examined the effect of apigenin on IFNα/β-induced JAK/STAT signaling. As shown in Fig. 4a, apigenin potently increased ISRE luciferase reporter expression induced by IFN-α or IFN-β in a concentration-dependent manner. Apigenin at 10 µM alone significantly increased ISRE reporter expression, as previously reported (17). We then examined the effect of apigenin alone on tyrosine phosphorylation of STAT1, the key factor mediating ISRE gene transcription. However, no obvious tyrosine phosphorylation of STAT1 was observed, possibly owing to the limitation of detection using anti-phospho-STAT1 antibody (data not shown). To further determine the effect of apigenin on type I IFNs, we examined the phosphorylation state of STATs in response to IFN-α. Apigenin increased the tyrosine phosphorylation of STAT1 and STAT2 in a concentration-dependent manner over that observed with IFN-α alone (Fig. 4b). The tyrosine phosphorylation of STAT1 was also increased in combination with luteolin and various concentrations of IFN-α (Supplementary Fig. 3). PKR and 2’,5’-OAS1 are IFN-α-responsive genes that contain an ISRE consensus sequence in their promoter regions. We examined the effect of apigenin on the mRNA expression of these two genes in combination with IFN-α. As shown in Fig. 4c, the mRNA expression of both IFN-regulated genes significantly increased after treatment with a combination of apigenin and IFN-α over that accompanying IFN-α treatment alone. These results indicate that apigenin enhances the activation of type I IFNs-induced JAK/STAT signaling.

### Apigenin inhibits the ubiquitination and degradation of IFNAR1

**Fig. 5:**
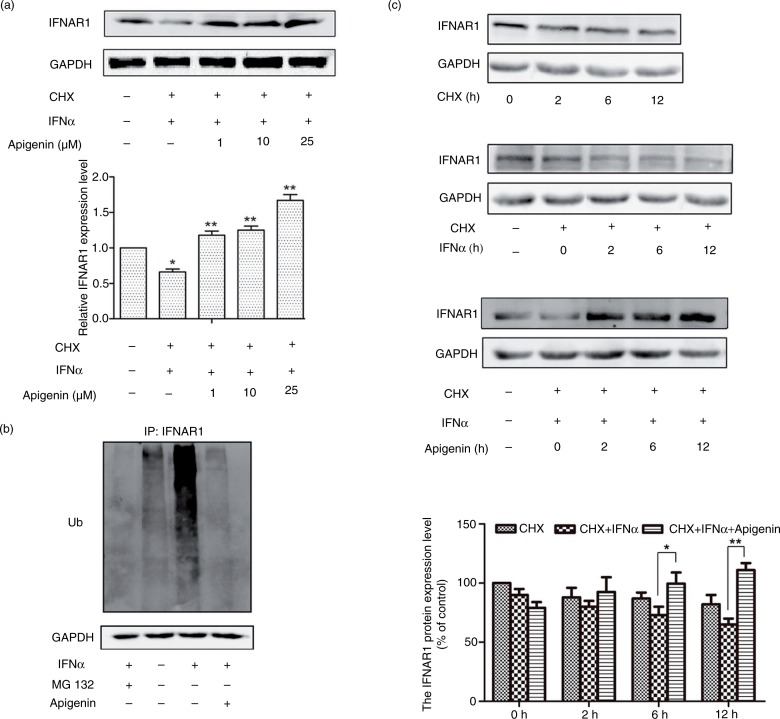
Apigenin inhibits IFN-α-induced degradation of IFNAR1. (a) HEK293A cells were treated with 20 µM cycloheximde (CHX) for 2 h, and then with apigenin (1 µM, 10 µM, 25 µM) for 12 h before the treatment with IFN-α (1×10^4^ U/mL) for an additional 2 h. The cell lysates were immunoblotted with anti-IFNAR1 antibody. GAPDH staining is shown as a loading control. (b) HeLa cells were transfected with pCMV-ubiquitin plasmid for 48 h and incubated with MG132 (20 µM) or apigenin (20 µM) for 12 h before treatment with IFN-α (1×10^4^ U/mL) for another 2 h. Cell lysates were immunoprecipitated with the IFNAR1 antibody. Immunoblotting was performed using ubiquitin antibody. GAPDH antibody staining represents 5% of the total cell lysates used in immunoprecipitation. (c) HeLa cells were treated with 20 µM CHX, IFN-α (1×10^4^ U/mL) or apigenin (20 µM) for the indicated time. The cell lysates were immunoblotted with anti-IFNAR1 antibodies. GAPDH staining is shown as a loading control.

Ligand-induced degradation of IFNAR1 is a key step in the negative regulation of IFN signaling. As an inhibitor of 26S proteasome, apigenin may activate the JAK/STAT pathway by inhibiting IFNAR1 degradation. To test this hypothesis, we examined the effect of apigenin on IFNAR1 degradation. As shown in Fig. 5a, in the presence of cycloheximde (CHX) to block *de novo* protein synthesis, IFN-α treatment stimulated the degradation of IFNAR1, as previously reported (13). Apigenin significantly inhibited the degradation of IFNAR1 stimulated by IFN-α. IFNAR1 is ubiquitinated for lysosomal degradation, which can be inhibited by 26S proteasome inhibitors (13). We next examined the effect of apigenin on IFNAR1 ubiquitination. We transiently overexpressed ubiquitin in cells and performed a co-immunoprecipitation experiment using the anti-IFNAR1 antibody. As shown in Fig. 5b, IFN-α treatment simulated the ubiquitination level of IFNAR1, which was inhibited by the 26S proteasome inhibitor MG132 and by apigenin. We also examined the effect of apigenin on ubiquitination level of endogenous proteins in the cell lysates transiently overexpressing ubiquitin. IFN-α slightly decreased ubiquitination level of endogenous proteins compared with DMSO control. Compared with that, addition of MG132 or apigenin increased ubiquitination level of endogenous proteins (Supplementary Fig. 4). We then examined the time course of apigenin in the inhibition of IFN-α-induced IFNAR1 degradation. As shown in Fig. 5c, apigenin significantly increased the expression of IFNAR1 in the presence of IFN-α in a time-dependent manner, compared with that in the presence of IFN-α alone. MG132 also inhibited IFN-α-induced IFNAR1 degradation in a time-dependent manner (Supplementary Fig. 5). These results indicate that apigenin protects IFNAR1 from degradation.

### Apigenin potentiates the inhibitory effect of IFN-α on cancer cell viability

**Fig. 6:**
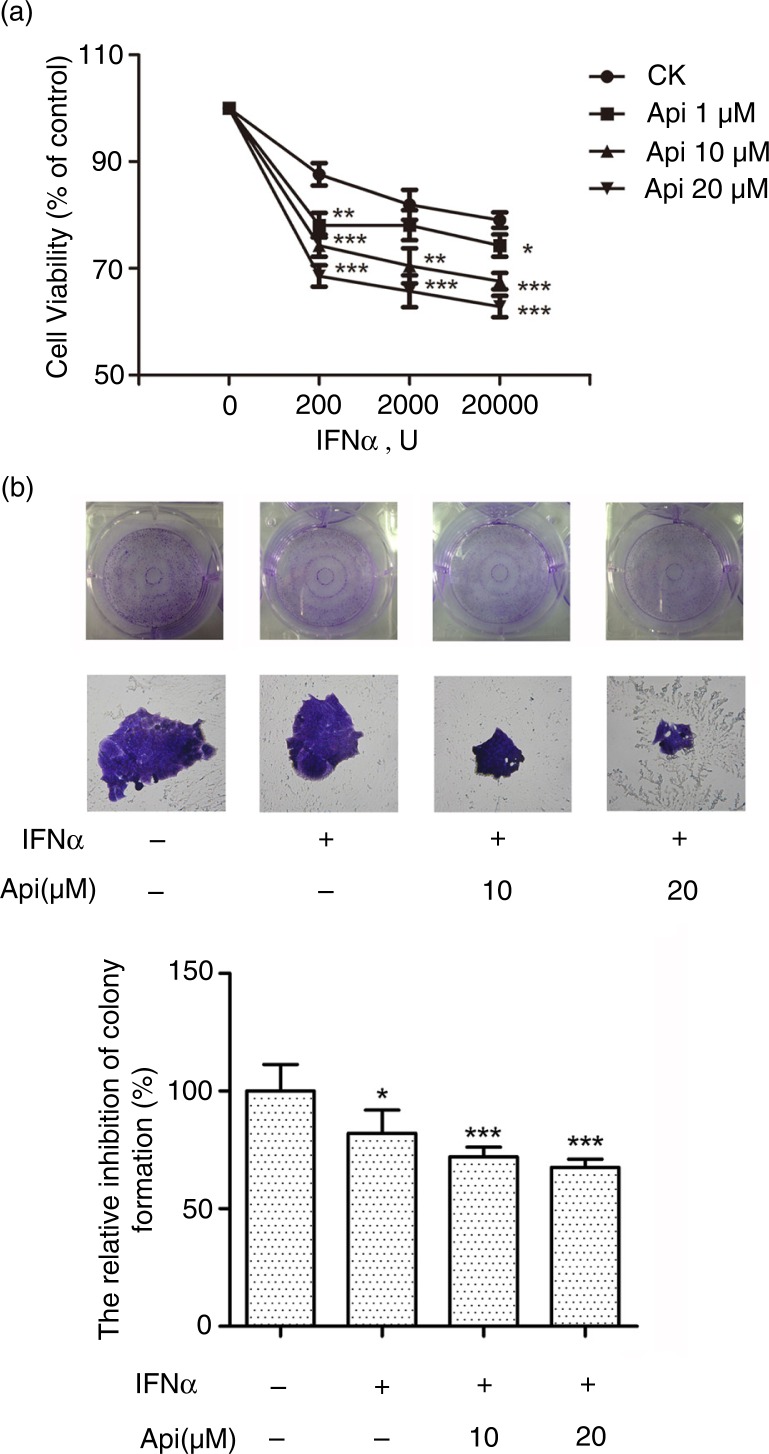
Apigenin potentiates the inhibitory effect of IFN-α on cancer cell viability. (a) The HeLa cells (5×10^3^ cells/well) were seeded in 96-well plates and treated under indicated concentrations of apigenin and IFN-α for 72 h. Cell viability was measured by Alamar Blue assay and the values are expressed as the percentage cell viability relative to the DMSO control. (b) The HeLa cells growing in 6-well plates were treated with the indicated concentrations of apigenin and IFN-α (1×10^4^ U/mL) for 12 days, and then colonies were visualized by staining with crystal violet and counted manually. The bar graph was obtained by calculating the percentages of colony numbers from each well relative to the DMSO-treated control. (*) *p*<0.05, (**) *p*<0.01, (***) *p*<0.001 versus control (*n=*3).

To examine whether apigenin can potentiate the inhibitory effect on cell viability induced by IFN, we treated human cervical cancer HeLa cells with apigenin and IFN-α. As shown in Fig. 6a, apigenin, in a concentration-dependent manner, significantly enhanced the inhibitory effect of IFN-α on cell viability, whereas apigenin alone at 1–20 µM had no effect on HeLa cell viability (Supplementary Fig. 6). Furthermore, INF-α alone or IFN-α plus apigenin did not exhibit the suppression on the cell viability of non-carcinoma HEK293A cells (Supplementary Fig. 7). We then examined the effect of apigenin on the proliferation and apoptosis of HeLa cells. As shown in Supplementary Fig. 8, the pro-apoptotic effects of INF-α alone or IFN-α plus apigenin were not observed. Moreover, treatment with IFN-α resulted in decreased colony formation in HeLa cells compared with the untreated control cells. The addition of apigenin significantly decreased the number and size of colonies, respectively, compared with cells treated by IFN-α alone (Fig. 6b). These results suggest that apigenin enhances the inhibitory effect of IFN-α on viability but not on proliferation and apoptosis in HeLa cancer cells.

## Discussion

By using a cell-based screening assay, apigenin was identified as an inhibitor of 26S proteasome-mediated luciferase-cODC degradation. This result is well supported by previous findings that apigenin inhibits the chymotrypsin-like activity of 20S proteasome and induces apoptosis in tumor cells, thus validating that the assay can be used for the screening and evaluation of 26S proteasome activity (2, 3) Apigenin has been found to inhibit, in a concentration-dependent manner from 1 to 20 µM, skin cancer promotion by directly inhibiting epidermal ODC activity induced by 12-O-tetradecanoyl phorbol-13-acetate (TPA) (22). In this study, apigenin was found to inhibit cODC-induced luciferase degradation at similar concentrations (1–20 µM), indicating that apigenin may not only inhibit ODC activity but also stabilize ODC protein. Apigenin inhibited the chymotrypsin-like activity of purified 26S proteasome with a potency different from that observed in previous studies; this difference was possibly owing to the fact that the proteasome used in these studies was from different species (2, 23). The specificity of apigenin for the three peptidase activities of human 26S proteasome was previously unknown. In this study, apigenin inhibited the three peptidase activities in the following order of potency caspase-like activity>chymotrypsin-like activity>trypsin-like activity. This result is consistent with the previous finding that flavones have strong inhibitory effects on chymotrypsin-like and caspase-like activities (23). Structure–proteasome inhibitory activity relationship studies of dietary flavonoids suggest that dietary flavonoids with OH groups on the B ring and/or the double bond between C2 and C3 of the pyranosyl moiety are natural potent proteasome inhibitors (24). This is further supported by our finding that apigenin could be favorably inserted within the S1 hydrophobic pockets of the β1, β2, and β5 subunits through H-bonding and hydrophobic interactions. The two hydroxyl groups on ring A of apigenin play a key role in the formation of H-bonds with residues in the active site of β1, β2, and β5 subunits to inhibit the Thr1 residue-mediated cleavage of peptides. These results also further support the finding that the 6-hydroxy group of the flavones may have an important role in targeting 26S proteasome, and this would help in the development of new 26S proteasome inhibitors (23).

Apigenin at high concentrations (>40 µM) induces apoptosis in leukemia cells and breast cancer cells and sensitizes the malignant tumor cells to tumor necrosis factor-related apoptosis-inducing ligand (TRAIL)-induced apoptosis (2–4). However, the daily dietary intake of apigenin is less likely to increase the concentration of apigenin to such high levels. This is the first study to report that apigenin at physiologically achievable concentrations (1–20 µM) can activate the type I IFN-induced JAK/STAT pathway and increase the endogenous IFN-regulated gene expression, resulting in the sensitization of cancer cells to the anti-cell viability effect of type I IFNs. In this study, we found that apigenin increased IFNAR1 accumulation in a time- and concentration-dependent manner, indicating that it may stabilize IFN-bound IFNAR1 via proteasome inhibition. Ubiquitination of IFNAR1 is required for IFNAR1 endocytosis and lysosomal degradation, similar with other cytokine receptors such as epidermal growth factor receptor and interleukin-2 receptor, which is mediated by 26S proteasome (14, 25, 26). Here, we found that IFN-α treatment could increase IFNAR1 ubiquitination, which was significantly inhibited by MG132 or apigenin. This result indicated that apigenin suppressed IFNAR1 degradation by inhibition of the 26S proteasome, similar to a previous finding that MG132 and lysosomal inhibitors promoted IFNAR1 accumulation (13). The manner in which inhibition of 26S proteasome activity decreases IFNAR1 ubiquitination is unknown. It is possible that inhibition of the 26S proteasome suppresses the activity of E3 ubiquitin ligase SCF^βTrcp^, which is responsible for IFNAR1 ubiquitination, or promotes the activity of an unknown deubiquitinase responsible for decreasing IFNAR1 ubiquitination (14, 27). Ligand-induced degradation of IFNAR1 is one of the key steps in the negative regulation of IFN signaling and is associated with the clinical efficacy of IFNs. Thus, protection of IFNAR1 from degradation in the presence of IFNs is proposed to be a new therapeutic means to enhance the efficacy of type I IFNs for the treatment of tumorigenesis, which requires higher doses of type I IFNs than those used for the treatment of viral diseases (28). In addition, apigenin is found to exhibit antiviral effects, but the role of JAK/STAT pathway in its inhibitory effects on viral replication is unknown (29–32). Here, for the first time we found that apigenin can inhibit the ubiquitination and degradation of IFNAR1, suggesting its potential as a new adjuvant for improving the efficacy of type I IFNs in the treatment of viral infections or cancers.

The antiviral and anticancer properties of phytoestrogens such as flavonoids have been widely reported, but their roles in the JAK/STAT pathway have not been studied in detail. Luteolin, another dietary flavonoid, has been found to inhibit 26S proteasome (24) and activate the type I IFNs-induced JAK/STAT pathway by enhancing cAMP-hydrolyzing activity of phosphodiesterases and decreasing intracellular cAMP levels for the suppression of PKA-mediated inhibition of protein tyrosine phosphatase SHP2 in cancer cells (12). This indicates that dietary flavonoids may sensitize the effects of type I IFNs through mechanisms other than the inhibition of 26S proteasome. Low levels of IFN-α/β are produced even in the absence of viral infection so as to maintain a constitutive weak IFN-α/β signal to elicit a rapid and strong cellular response against infection (33). Various flavonoids show additive antiproliferative activity with IFN-α via an unknown mechanism (34). This study provides new insights into how phytoestrogens exert their antiviral effects and their possible roles in potentiating the antiviral and anticancerous JAK/STAT signaling pathway. Apigenin is abundant in common fruits and vegetables, including parsley, onions, oranges, tea, chamomile, wheat sprouts, and some seasonings. In clinical trials, apigenin has shown good safety (35–37). In this study, apigenin was used at concentrations previously reported to translate into well-tolerated and active human serum concentrations (35–37). Thus, to a certain extent, the old saying that ‘an apple a day keeps the doctor away’ is indeed true because the dietary flavonoids keep the virus at bay through the synergistic action with the type I IFNs (38).

In conclusion, we found that apigenin, distributed widely in fruits and vegetables, potentiates the inhibitory effects of type I IFNs on cancer cell viability by the activation of JAK/STAT pathway through the inhibition of 26S proteasome and stabilization of IFNAR1. Apigenin warrants further investigation as a potential adjuvant for type I IFNs therapy in the treatment of viral or cancerous diseases. Consumption of fruits and vegetables rich in apigenin could prevent viral infection and reduce tumorigenesis.

## Supplementary Material

Dietary apigenin potentiates the inhibitory effect of interferon-α on cancer cell viability through inhibition of 26S proteasome-mediated interferon receptor degradation
