# Supplementary material for: Dietary apigenin potentiates the inhibitory effect of interferon-α on cancer cell viability through inhibition of 26S proteasome-mediated interferon receptor degradation
Source: Food Nutr Res. 2016 Jun 28;60:10.3402/fnr.v60.31288. doi: 10.3402/fnr.v60.31288 (PMC4928072; doi:10.3402/fnr.v60.31288)
Supplement: Dietary apigenin potentiates the inhibitory effect of interferon-α on cancer cell viability through inhibition of 26S proteasome-mediated interferon receptor degradation [file FNR-60-31288-s001.doc]

**Supplementary Materials**


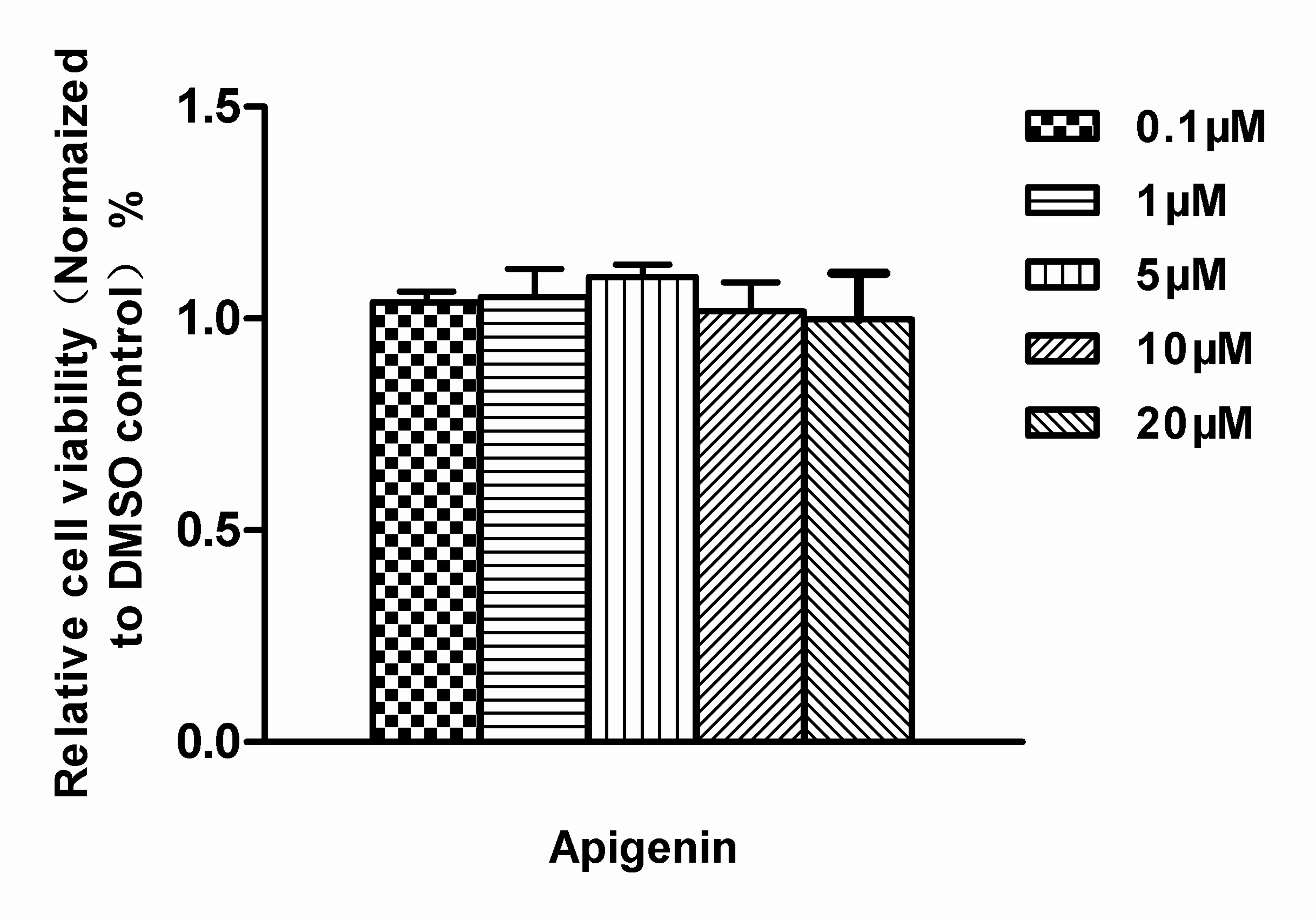


**Fig. S1. Cytotoxicity of apigenin in HEK293A-luciferase-cODC cells**. Cells were treated with apigenin (0.1, 1, 5, 10 and 20 μM) for 24 h, and then the cell viability was examined by Alamar-Blue assay. Values are mean ± s.d. for triplicate samples after normalization to DMSO control.


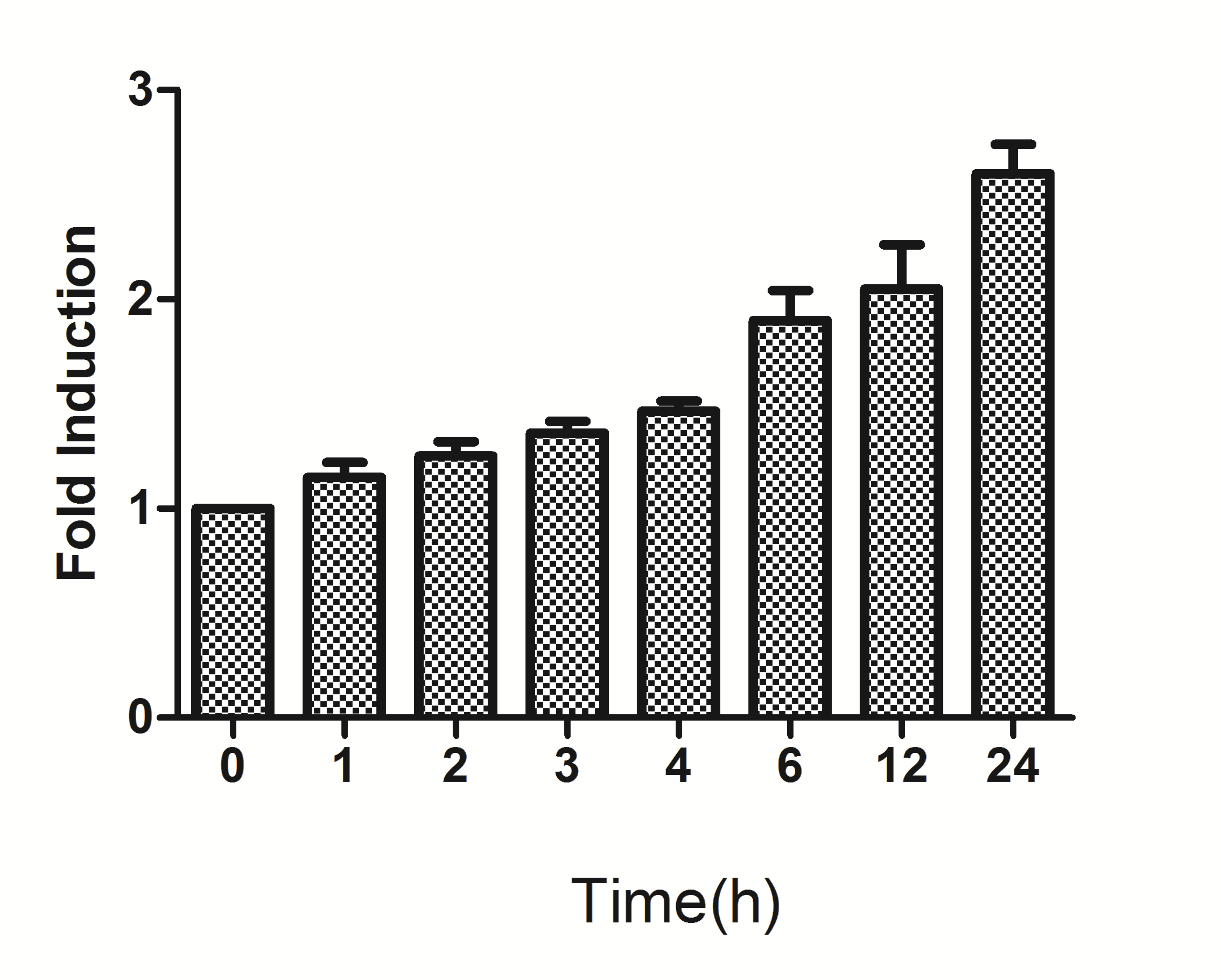


**Fig. S2. Effect of MG132 on luciferase-cODC expression**. HEK293A-luciferase-cODC cells were seeded into a 96-well plate and treated with MG132 (1 μM) for different time. The cells were lysed and luciferase activity was measured.


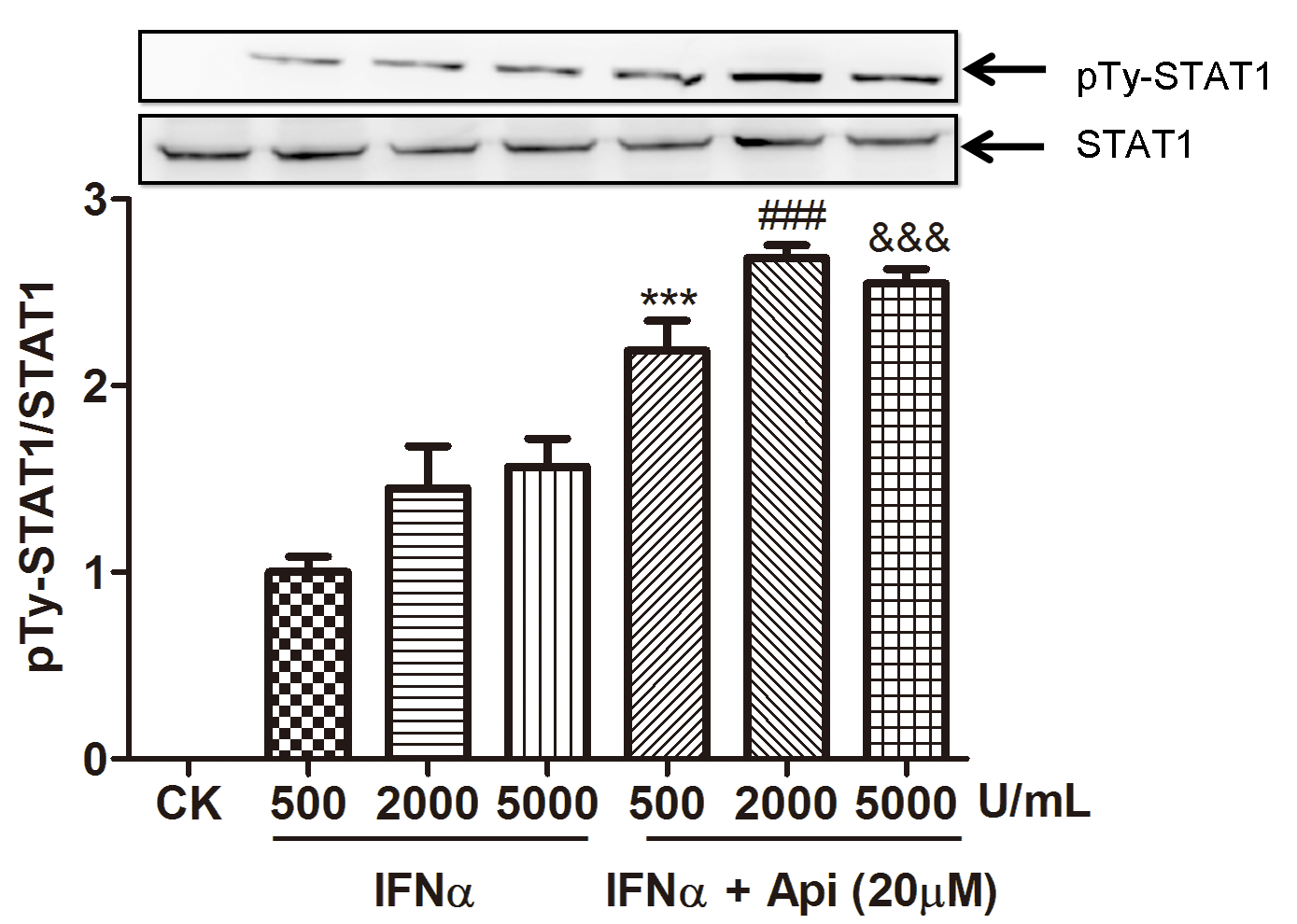


**Fig. S3. Effect of IFN-α on the tyrosine phosphorylation of STAT1.** HEK293A cells were incubated with indicated concentrations of apigenin for 2 h, then with indicated concentrations of IFN-α for another 1 h. The cell lysates were immunoblotted with phospho-STAT1 (Tyr701) and STAT1 antibodies. The quantitative results are shown. (***) *p* < 0.001 vs. 500U/mL of IFN-**α**; (###) *p* < 0.011 vs. 2000U/mL of IFN-**α;** (&&&) *p* < 0.001 vs. 5000U/mL of IFN-**α,** *n*=3.


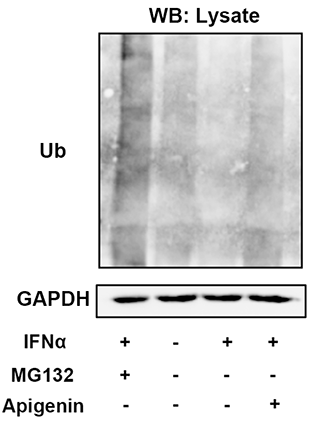


**Fig. S4.** **Effect of apigenin on ubiquitination level of endogenous proteins in the presence of IFN-α**. HeLa cells were transiently transfected with pCMV-ubiquitin plasmid for 48 h, and then were incubated with MG132 (20 μM) or apigenin (20 μM) for 12 h，followed by the addition of IFN-α (1×10^4^ U/mL) for another 2 h. Immunoblotting was performed using anti-ubiquitin antibody. GAPDH antibody staining represents 5% of the total cell lysates used in immunoprecipitation.


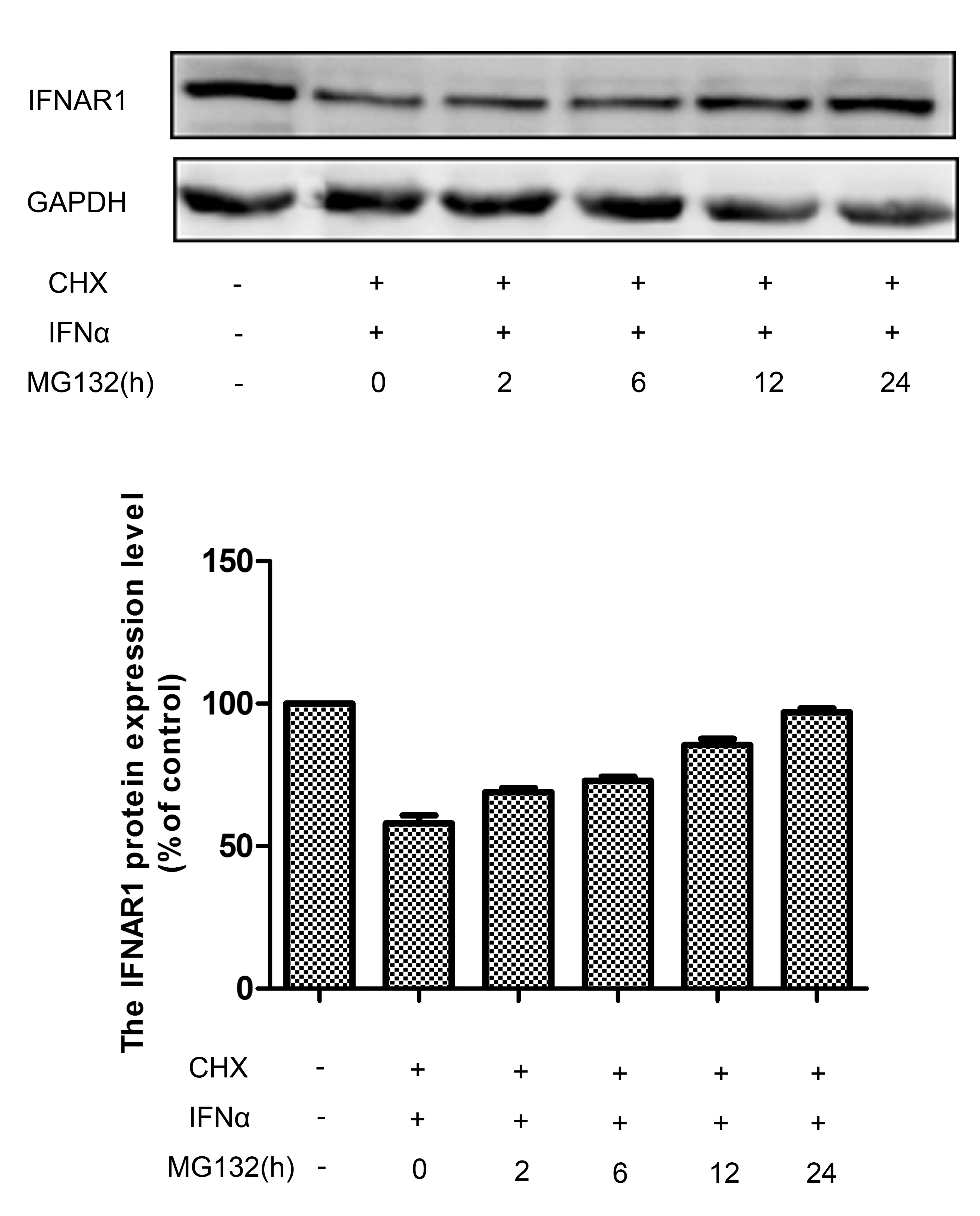


**Fig. S5. Effect of MG132 on IFNAR1 degradation.** HeLa cells were treated with 20 μM cycloheximide (CHX) for 2 h, followed by the addition of MG132 (20 μM) for 0-24 h and treatment with IFN-α (1 × 10^4^ U/mL) for 2 h. The IFNAR1 expression level was detected by western blotting. Quantitative result is described as a histogram.


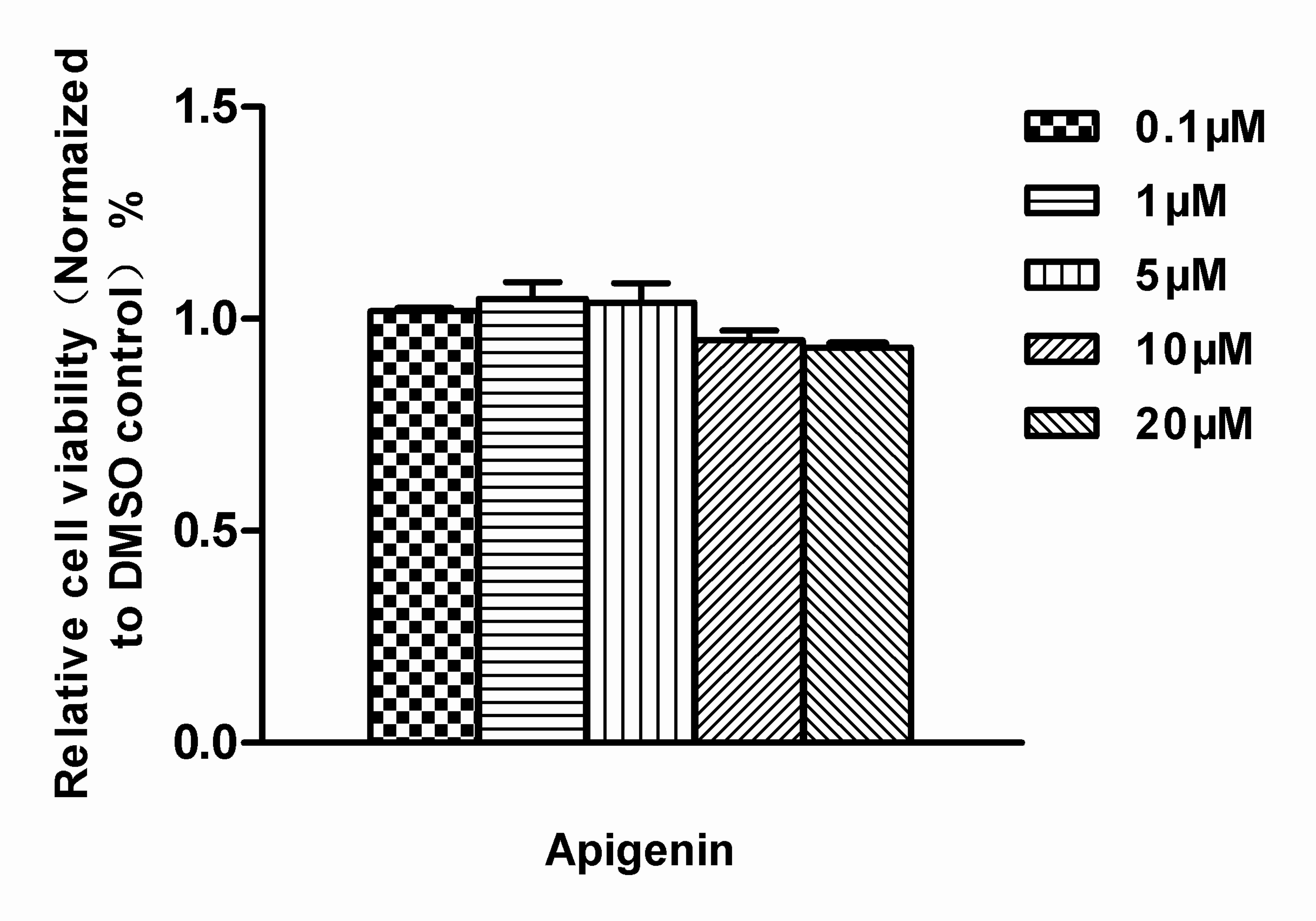


**Fig. S6. Cytotoxicity of Apigenin in HeLa cells**. Cells were treated with Apigenin (0.1, 1, 5, 10 and 20 μM) for 72 h, and then the cell viability was examined by Alamar-Blue assay. Values are mean ± s.e.m. for triplicate samples after normalization to DMSO control.

**
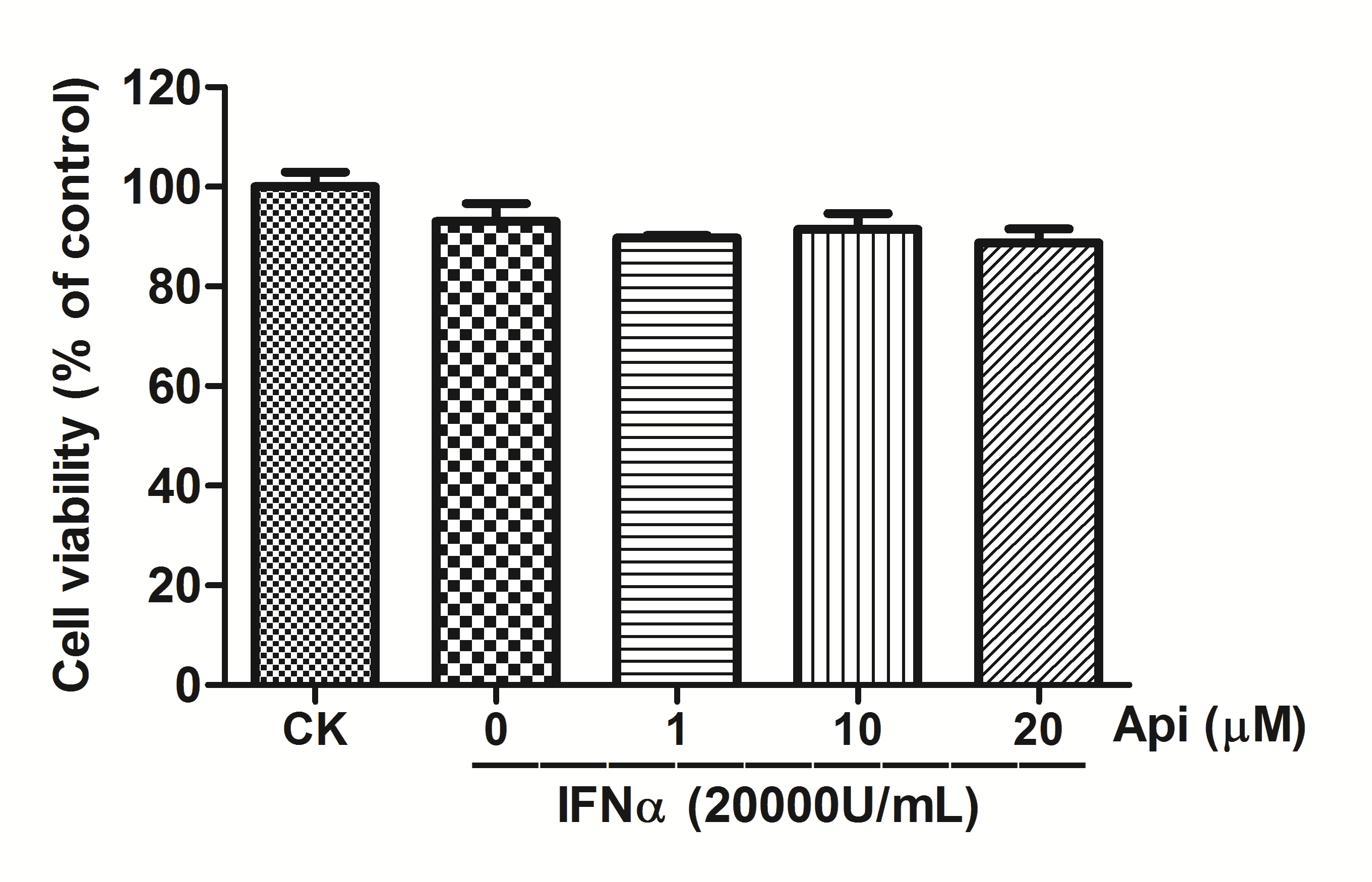
**

**Fig. S7. Cytotoxicity of Apigenin plus IFN-α in HEK293A cells.** The 293A cells (5×10^3^ cells/well) were seeded in 96-well plates and treated under indicated concentrations of apigenin and IFN-α for 72 h, and then the cell viability was examined by Alamar-Blue assay. Values are mean ±s.e.m. for triplicate samples after normalization to DMSO control.

**
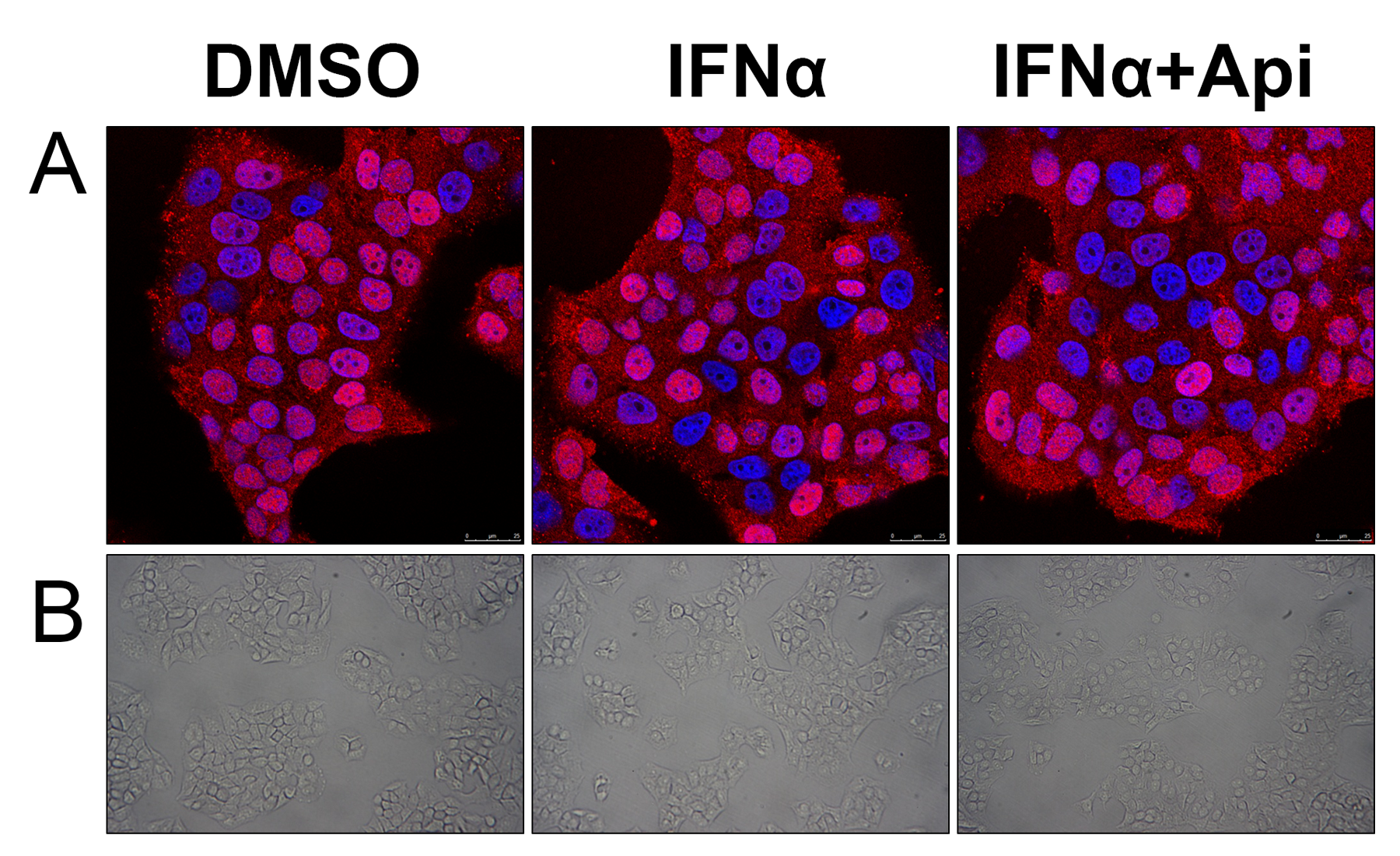
**

**Fig. S8. Effect of Apigenin plus IFN-α on the proliferation and apoptosis in HeLa cells.** (A) HeLa cells (1×10^5^ cells/well) were seeded in 35 mm culture dishs and treated under indicated concentrations of apigenin and IFN-α for 72 h. The cells were fixed, permeabilized, and processed for immunofluorescence using KI67 antibody and Alexa Fluor 555-labeled anti-rabbit IgG (red, positive signal) as well as DAPI (blue, nucleus). (B) After apigenin and IFN-α treatment, HeLa cells were fixed, permeabilized, and incubated with terminal deoxynucleotidyl transferase and biotin-labbed deoxyuridine triphosphate, as well as streptavidin-HRP, respectively. Apoptotic cells were visualized by DAB reagent.
